# A 10-Year Single-Center Experience With the GORE TAG Conformable Thoracic Stent Graft in the Treatment of Thoracic Aortic Disease

**DOI:** 10.1177/15266028211049340

**Published:** 2021-10-11

**Authors:** Denis Skrypnik, Moritz S. Bischoff, Katrin Meisenbacher, Dorothea B. Kronsteiner, Dittmar Böckler

**Affiliations:** 1 Department of Vascular and Endovascular Surgery, University Hospital Heidelberg, Heidelberg, Germany; 2 Institute of Medical Biometry and Informatics, Heidelberg University, Heidelberg, Germany

**Keywords:** aorta, TEVAR, outcome, survival, reintervention

## Abstract

**Objective:**

The aim of this study was to report 10-year real-world single-center experience with the GORE TAG conformable thoracic aortic graft (CTAG), focusing on rupture-free survival, aortic-related reintervention, and device-related complications during midterm and long-term follow-up (FU).

**Methods:**

This retrospective study analyzes results of thoracic endovascular aortic repair (TEVAR) performed between January 2009 and December 2018. Out of 419 TEVAR procedures within this period, 194 patients (male 57.2%, 111/194), with a mean age of 65 ± 13 years, were treated with the CTAG device. Indication for TEVAR was a thoracic aortic aneurysm in 24.7% (48/194), type B aortic dissection in 32.5% (63/194), penetrating aortic ulcer 15.5% (30/194), and miscellaneous 27.3% (53/194). Emergently were operated 43.8% (85/194) patients. Median follow-up (FU) including computed tomography imaging was 43.5 months (Q1-Q3: 8.6–67.0) and was completed in 91.2% (177/194) of patients.

**Results:**

Overall survival rates were 75.8% (95% confidence interval [CI] = [0.76–0.70]) and 56.6% (95% CI = [0.57–0.50]) at 12 and 60 months, respectively. Cumulative incidence for aortic rupture was 11.9% (95% CI = [0.07–0.17]) at 60 and 90 months, respectively. Cumulative incidence for aortic-related reintervention was 27.5% (95% CI = [0.21–0.34]) at 60 and 90 months. Cumulative incidence for migration was 2.8% (95% CI = [0.004–0.05]) and 3.9% (95% CI = [0.007–0.07]) at 60 and 90 months, respectively. New endograft infections or material fatigue were not observed.

**Conclusions:**

The herein reported 10-year real-world single-center experience with the CTAG observed favorable long-term outcome. Thus, the device demonstrates appropriate persistent safety, efficacy, and clinical durability up to long-term FU in the treatment of diverse thoracic aortic pathologies.

## Introduction

Thoracic endovascular aortic repair (TEVAR) is the standard for the treatment of various thoracic aortic pathologies in most centers worldwide.^1–3^ Anatomical challenges of thoracic aorta and distal aortic arch require from thoracic endografts appropriate safety, efficacy, and clinical durability, as well as freedom from device-related complications (DRCs).

The GORE TAG *c*onformable *t*horacic *a*ortic *g*raft (CTAG; WL Gore & Assoc., Flagstaff, AZ, USA) was initially designed for the treatment of aortic arch. Compared with the first generation of the Gore stent graft (TAG), the CTAG has undergone several modifications to adapt the radial force and to improve aortic wall apposition in challenging aortic anatomies.^4,5^ Previously published clinical trials have reported favorable short-term and midterm results for the use of CTAG to treat thoracic aortic aneurysm (TAA), type B aortic dissections (TBAD), and traumatic aortic rupture (TAR).^6–8^

The durability of CTAG was reported in a European post-market multicenter single-arm study with a maximum FU of 2.7 years.^9^ The CTAG dissection study reported a maximum FU period of 3.5 years, wherein 30% (15/50) of patients were followed up for more than 36 months.^7^

With long-term data lacking, the aim of this study was to report 10-year real-world single-center experience with CTAG, focusing on rupture-free survival, aortic-related reintervention (ARR), and DRCs in the midterm and long-term FU period.

## Methods

### Study Design

A single-center retrospective analysis of prospectively collected clinical observational data and computed tomography (CT)–based imaging data was performed. Informed consent for processing and collection of clinical and morphological data was obtained for each case following the principles of the Declaration of Helsinki. Ethical approval from the local ethics committee for use of the prospectively collected TEVAR database was obtained (Protocol No. S-158/2015).

### Study Population

**Figure 1.**
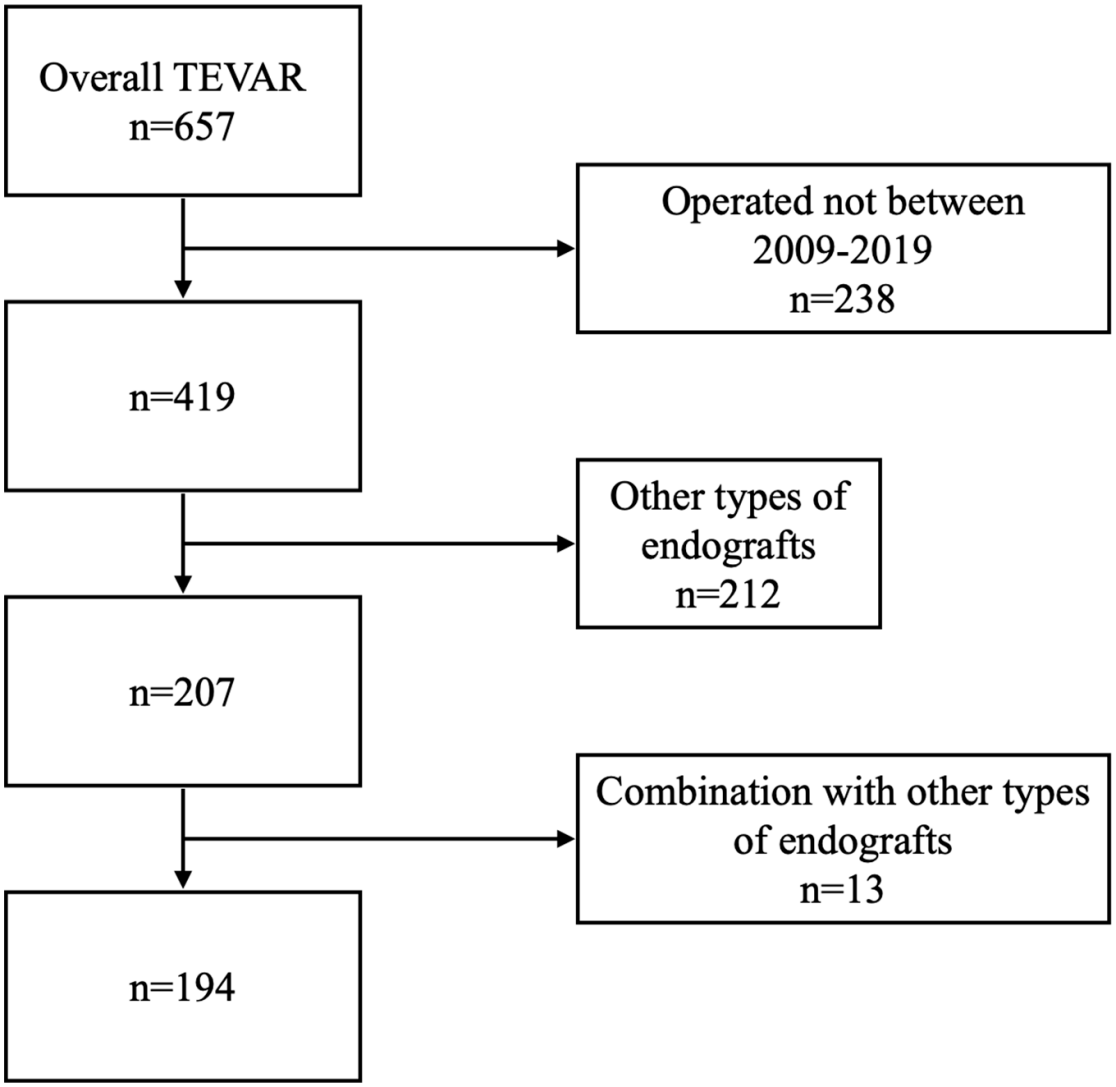
Flowchart. TEVAR, thoracic endovascular aortic repair.

In 194 out of 419 TEVAR procedures, performed between January 1 2009, and December 31, 2018, the CTAG device was implanted (Figure 1). The study enrolled patients with TAA, thoracoabdominal aortic aneurysm (TAAA), TBAD, penetrating aortic ulcer (PAU), intramural hematoma (IMH), aortoesophageal fistula (AEF), aortobronchial fistula (ABF), TAR, and anastomotic or patch aortic aneurysm who were treated with TEVAR using the CTAG (WL Gore & Assoc.). Patients who received any other type of endograft (n = 212), and endograft combinations or Gore Conformable Thoracic Stent Graft with Active Control System (n = 13) were excluded (Figure 1). The demographic, anatomical, periprocedural, and longitudinal data were prospectively collected in line with current reporting standards.^10^

The study included 194 patients, predominantly males (57.2%, 111/194), with a mean age of 65 ± 13 years. Arterial hypertension and a history of smoking were the most common risk factors reported (82% and 51%, respectively). Previous abdominal or thoracic aortic surgery was present in 28% of the cases. Patient demographics are displayed in Table 1. TAA and aortic dissection were the most frequent indications for TEVAR. A detailed description of the indications for treatment and the TEVAR settings is presented in Table 2.

**Table 1.** Patient Demographic Data.

Age	65 ± 13.4
Male	57.2 (111/194)
Female	42.8 (83/194)
BMI, kg/m^2^	25.7 ± 7.1
ASA^a^	
0	0.5 (1/192)
1	1.0 (2/192)
2	10.4 (20/192)
3	63.0 (121/192)
4	24.5 (47/192)
5	0.5 (1/192)
Hypertension^a^	82.3 (158/192)
Coronary heart disease	28.3 (55/194)
Heart insufficiency^b^	10.5 (20/190)
Previous MI	9.7 (19/194)
Carotid stenosis	8.7 (17/194)
Previous stroke	3.1 (6/194)
PAD^c^	8.9 (17/191)
COPD	13.4 (26/194)
Diabetes	12.4 (24/194)
Adipositas (BMI > 30)	21.1 (41/194)
Chronic renal insufficiency (creatinine > 1.2 mg/dl)	14.9 (29/194)
History of smoking^d^	51.0 (96/188)
Previous aortic surgery^e^	28.1 (54/192)

Categorical data are presented as absolute numbers and percentage; continuous data are presented in mean ± SD (n = 194).

Abbreviations: ASA, American Society of Anesthesiologists; BMI, body mass index; COPD, chronic obstructive pulmonary disease; MI, myocardial infarction; PAD, peripheral arterial disease.

a n = 2 patients’ data were not accessible.

b n = 4 patients’ data were not accessible.

c n = 3 patients’ data were not accessible.

d n = 6 patients’ data were not accessible.

e n = 2 patients’ data were not accessible.

**Table 2.** Indications for Treatment and Procedure Settings.

Diagnosis	
TAA/TAAA	20.6 (40/194)
rTAA	4.1 (8/194)
TBAD/rest-TAAD	24.7 (48/194)
CEAD	7.7 (15/194)
PAU	15.5 (30/194)
IMH	9.3 (18/194)
TAR	8.2 (16/194)
Aortobronchial/aortoesophageal fistula	5.2 (10/194)
Anastomotic aneurysm	4.1 (8/194)
Other^a^	0.5 (1/194)
Malperfusion^b^	8.2 (16/194)
Type of aortic arch^c^	
1	24.3 (46/189)
2	46.7 (88/189)
3	29.1 (55/189)
Grade of aortic arch atheroma^c^	
0–1	47.1 (89/189)
2	18.5 (35/189)
3	21.7 (41/189)
4–5	12.7 (24/189)
Type of PLZ	
0–1	9.8 (19/194)
2	34.0 (66/194)
3	33.5 (65/194)
4^d^	21.1 (41/194)
5	1.5 (3/194)
Multiple devices	38.7 (75/1194)
Emergency operation	43.8 (85/194)
General anesthesia	92.2 (179/194)
Spinal drainage	36.4 (70/192)^e^
Access	
Transfemoral	74.2 (144/194)
Iliac conduit	6.2 (12/194)
Coverage of LSA	42.3 (82/194)
Primary revascularization of LSA	19.1 (37/194)
Secondary revascularization of LSA	3.6 (7/194)
Hybrid procedures^f^	25.8 (50/194)
Chimney^g^	4.1 (8/194)

Data are presented as percentage (n = 194).

Abbreviations: CEAD, chronic expanding aortic dissection; IMH, intramural hematoma; LSA, left subclavian artery; PAU, penetrating aortic ulcer; PLZ, proximal landing zone; rest-TAAD, rest–type A aortic dissection; rTAA, ruptured TAA; TAA, thoracic aortic aneurysm; TAAA, thoracoabdominal aortic aneurysm; TAR, traumatic aortic rupture; TBAD, type B aortic dissection.

a Intercostal artery aneurysm.

b Renal, visceral, lower extremity, spinal cord, or combined malperfusion.

c n = 5 patients’ data were not accessible.

d Proximal landing zone in “frozen elephant trunk” included.

e n = 2 patients’ data were not accessible.

f Aortic arch debranching (carotid subclavian/carotid carotidal bypass, carotid subclavian transposition, carotid subclavian bypass with implantation of left carotid artery in the bypass) and/or visceral debranching (aortic hepatic bypass, total visceral debranching).

g Chimney endograft of *Arteria mesenterica superior* and/or renal artery and/or *Truncus coeliacus* and/or left common carotid artery.

### Device Specifications

The CTAG consists of a self-expandable frame covered by a polytetrafluoroethylene sealing cuff. The proximal end of the endograft has a partially uncovered strut-crown for better wall apposition. The distal graft end is covered to avoid perforation of the dissection membrane. The structure of the body of the endograft is designed for better angulation with a low spring back force, dedicated to the treatment of aortic arch pathologies in aortic zones II–IV. The portfolio of the device proposes endovascular sealing with up to 42 mm aortic diameter and an oversizing range of 0% to 30% depending on the underlying pathology. A detailed description of the delivery system and deployment are available online (https://www.goremedical.com/products/ctag).

### Procedural Data

All operations were performed by certified vascular surgeons in hybrid operating rooms equipped with the Siemens Artis Zeego angiography system (Siemens Healthcare GmbH, Forchheim Erlangen, Germany). The institutional implantation protocol for TEVAR has been previously published.^11^

Of the 194 procedures, 92% (n = 179) were performed under general anesthesia (Table 2). Due to the high risk of paraplegia from aortic coverage >20 cm, coverage of left subclavian artery (LSA) in emergencies or previous infrarenal aortic repair, cerebrospinal fluid drainage (CSFD) was selectively applied with subsequent automatic intrathecal pressure control for at least 3 days.^12^

In 77% of cases, TEVAR was performed in arch zones 0 to 3. Of the procedures, 44% (85/194) were emergent and 26% (50/194) were accompanied by visceral or arch debranching (Table 2). Coverage of LSA was performed in 42% (82/194) of cases. Primary revascularization of LSA was carried out in 19% (37/194) of patients (Table 2).

### Imaging and FU

All patients received preoperative high-resolution computer tomographic angiography (CTA) of the entire aorta with 1 mm slice acquisition, including the arterial, venous, and delayed venous phase. The CTA was performed preoperatively, before discharge, at 6 months postoperatively and annually thereafter. The FU CT–Angiography analysis was performed using certified software-based centerline 3D (three-dimensional) reconstruction (Aycan Workstation OsiriX PRO; Aycan Medical Systems, Rochester, NY, USA). All radiological series were assessed using centerline 3D reconstructions by at least 2 independent vascular surgeons.

The FU was completed up to August 2020 for 91.2% (177/194) of patients. The missing 8.7% (17/194) of patients have changed their living place and contact details, and thus were declared lost to FU.

### Definitions

The endpoints of the study were rupture-free survival, ARR, and DRCs in the midterm and long-term FU period.

Aortic-related reintervention was defined as any endovascular, open, or hybrid procedure related to complications of aortic pathology, progression of aortic disease, or complications of TEVAR, including early and late conversion.

Primary technical success was defined according to the current reporting standards for TEVAR.^10^

Aortic-related reintervention was defined as any endovascular, open, or hybrid procedure related to complications of aortic pathology, progression of aortic disease, or complications of TEVAR, including early and late conversion.

Short-term FU was defined as the period after discharge up to 1 year postoperative, the midterm FU was defined as between 1 and 5 years postoperative, and long-term FU thereafter.^10^

The procedure-related systemic complications (PRSCs) were defined as stroke, paraplegia, myocardial infarction, acute renal failure, or colonic ischemia.^13^

The classification of aortic arch types, endoleaks (ELs), and landing zones was performed according to the previously published literature.^14–16^

Endoleaks were classified as primary if they were diagnosed intraoperatively, and secondary if they were detected during FU.^16^

Thrombogenicity of the aortic arch was graduated as “normal,” “intimal thickening,” atheroma <5 mm, atheroma >5 mm, and “mobile atheroma,” according to Feezor et al.^17^

Migration was defined as an endograft displacement of >10 mm relative to a primary anatomic landmark or any displacement that led to symptoms or required therapy during FU.^10^

The DRCs were defined as endograft migration, infection, material degradation, and endograft obstruction, as well as retrograde aortic dissection or IMH.^13^

Periprocedural stroke was defined as a focal or global loss of neurological function lasting more than 24 hours or leading to death, combined with vascular etiology.^18^

### Statistical Analysis

Patient and disease characteristics are presented as mean and standard deviation for continuous variables and absolute and relative frequencies for categorical variables. Median FU was given by the median including the 25% and the 75% quantiles (Q1-Q3). The Kaplan-Meier method is used to estimate the survival function for overall survival. Cumulative incidence functions (CIFs) were used for ARR, migration, and rupture to account for the competing risk death. The Kaplan-Meier curves and CIFs for the endpoints are stratified by pathology and by urgency of the procedure. The log-rank test was used to compare overall survival. Gray’s test was used to compare ARR, migration, and rupture of different pathologies, as well as the CIF according to the urgency of the procedure. Due to the explorative nature of this study, the presented p values are of descriptive nature. Statistical analyses were performed using R software (version 4.0.5, R Foundation for Statistical Computing, Vienna, Austria).

## Results

### In-Hospital Outcomes

#### Primary technical success

Primary technical success was achieved in 94.3% (183/194) of cases. Reasons for technical failure are listed below.

Four patients died within 24 hours after TEVAR due to hemorrhagic shock from primary rupture of TAA (n = 2), false lumen of TBAD, or bleeding from AEF.

One patient died due to high deceleration polytrauma and 1 due to multiorgan failure (MOF) due to complicated acute TBAD with multiorgan ischemia <24 hours after TEVAR.

One case of unintended intraoperative proximal endograft migration with partial overstenting of the left common carotid artery was treated with a crossover carotid-carotid bypass.

Due to complex aortic diameter mismatch after 2 open reoperations because of aortic isthmus stenosis, 1 type Ia EL was secondarily treated with proximal extension and debranching.

One primary type III EL was successfully treated with endolining during a secondary procedure, which was performed due to insufficient overlap.

One retrograde dissection was diagnosed. The patient was treated conservatively due to the high risk of cardiac surgery and died on the 5th postoperative day due to MOF.

One patient had retrograde IMH and was treated by open surgery; however, the patient died on postoperative day 5 due to myocardial infarction related to coronary bypass occlusion.

No intraoperative conversions were needed.

#### Mortality

The in-hospital all-cause mortality was 12.8% (25/194), of which the majority were due to MOF associated with hemorrhagic shock resulting from free rupture of TAA or TBAD (2.6%, 5/194) or due to bleeding from ABF or AEF (2.6%, 5/194; Table 3). Also, 5 patients (2.6%, 5/194) died due to MOF following visceral and lower limb malperfusion. Three lethal cardiac events (1.5%, 3/194) were due to progression of cardiac failure, myocardial infarction, and ventricular tachyarrhythmia. Acute respiratory failure, polytrauma, and sepsis were rarely presented (Table 3). One patient with gastrointestinal bleeding and 1 with venous bleeding due to full anticoagulation during extracorporeal membrane oxygenation (ECMO) were related to non-aortic lethal episodes.

**Table 3.** Cumulative Cause of Mortality.

Causes of death	Overall n = 83	In-hospital n = 25	Short term n = 20	Midterm n = 32	Long term n = 6
Rupture	5.2 (10)	2.6 (5)	0.5 (1)	1.5 (3)	0.5 (1)
Bleeding due to ABF/AEF	4.6 (9)	2.6 (5)	0.5 (1)	1.5 (3)	
Multiorgan failure	4.6 (9)	2.6 (5)	1.0 (2)	0.5 (1)	0.5 (1)
Cardiac events^a^	3.1 (6)	1.5 (3)		1.5 (3)	
Malignancy	4.6 (9)		1.5 (3)	3.1 (6)	
Acute respiratory failure	3.1 (6)	0.5 (1)	1.5 (3)	0.5 (1)	0.5 (1)
Stroke	2.6 (5)		2.1 (4)	0.5 (1)	
Sepsis	2.1 (4)	1.0 (2)	1.0 (2)		
Trauma	1.5 (3)	1.0 (2)	0.5 (1)		
Mesenteric ischemia/ileus	1.0 (2)			1.0 (2)	
Other^b^	1.0 (2)			1.0 (2)	
Non-aortic-related bleeding	1.0 (2)	1.0 (2)			
Unclear	8.2 (16)		1.5 (3)	5.2 (10)	1.5 (3)

Data are presented as percentage and absolute numbers (n = 194). ABF, aortobronchial fistula. AEF, aortoesophageal fistula.

a Myocardial infarction, hemodynamic relevant cardiac rhythm abnormalities, cardiac failure.

b Lung artery emboly, amyotrophic lateral sclerosis.

#### Procedure-related systemic complications

The PRSCs occurred in 34 cases (17.5%, 34/194; Table 4). Five patients suffered a postoperative stroke (2.6%, 5/194). All strokes were reported in patients who underwent TEVAR in proximal landing zone (PLZ) I, but none resulted in mortality (Table 4).

**Table 4.** In-Hospital Procedure-Related Systemic Complications.

Stroke	2.6 (5)
Paraplegia	4.1 (8)
Myocardial infarction	2.1 (4)
Acute renal failure	6.2 (12)
Colonic ischemia	2.6 (5)
Overall	17.5 (34/194)

Categorical data are presented as absolute numbers and percentage (n = 194).

Paraplegia occurred in 8 cases (4.1%, 8/194; Table 4). Seven patients were operated on urgently due to ruptured TAA or TAAA or complicated TBAD, in whom paraplegia was due to shock and/or true lumen collapse. One patient operated on for PAU showed secondary development of paraplegia during the postoperative period. In the last case, the neurological deficit was resolved following spinal drainage application. Paraplegia was persistent in 4 patients. Three patients died on postoperative days 3, 5, and 15 due to rupture of TAAA and complicated TBAD.

Colonic ischemia occurred in 5 cases (2.6%, 5/194; Table 4). Two patients were operated on due to complicated TBAD with a true lumen collapse resulting in visceral ischemia. Two patients had colonic ischemia due to shock associated with AEF and ruptured TAAA. One patient treated for TAAA showed occlusion of the visceral debranching bypass which resulted in colonic ischemia and death. In every patient except the latter, a colectomy was performed and no in-hospital mortality was observed.

Four patients had myocardial infarction (2.1%, 4/194) and 12 patients were complicated with acute renal failure (6.2%, 12/194) (Table 4).

#### Device-related complications

Two patients died due to retrograde aortic dissection and retrograde IMH related to DRC. Ten in-hospital endograft infections occurred (5.2%, 10/194) related to TEVAR in primary septic condition due to ABF or AEF. Two stent graft–induced new entry (SINE) occurred (1.0%, 2/194).

### Midterm and Long-Term Outcomes

#### Follow-up

The median FU was 43.5 months (Q1-Q3: 8.6–67.0 months). The longest FU was 127.8 months. By August 2020, FU was completed in 91.2% (177/194) of patients. In our study, 8.7% (17/194) of patients were lost to FU.

**Figure 2.**
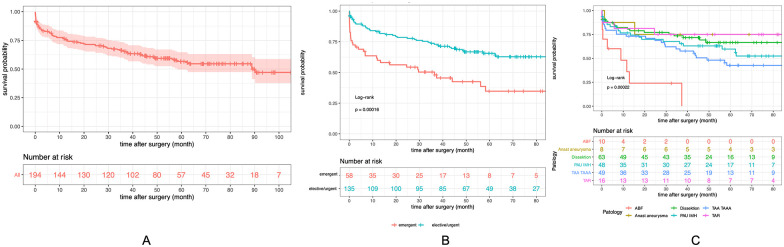
(A) Overall survival of all patients included in the study (Kaplan-Meier). Kaplan-Meier survival curve stratified by (B) emergent compared with elective/urgent patients, and (C) indication. ABF, aortobronchial fistula; AEF, aortoesophageal fistula.

In the study group, 72.2% (140/194) of patients were followed 12 months after TEVAR, and 29.4% (57/194) were followed over 60 months after TEVAR (Figure 2A).

#### Mortality

In the midterm FU, all-cause mortality was 16.5% (32/194). Three aortic ruptures were reported (1.5%, 3/194). One patient primarily treated for TBAD showed rupture of an ascending aorta aneurysm 19 months after TEVAR. One rupture occurred at 37 months in a patient who underwent TEVAR in PLZ 2, related to expansion of the aortic arch without EL. One rupture was due to endograft migration 43 months after TEVAR, related to TAA expansion due to persistent type II EL via the intercostal arteries.

Three patients operated on due to ABF or AEF died during FU due to aortic septic hemorrhage. Six patients (3.1%, 6/194) died due to malignancy. One stroke occurred 17 months after TEVAR but was not associated with TEVAR. Three lethal cardiac events were due to myocardial infarction and progression of cardiac failure.

In the long-term FU, all-cause mortality was 3.1% (6/194). Aortic rupture was due to migration, diagnosed 64 months after TEVAR due to TAA progression. The patient denied reintervention and died 91 months after TEVAR. One MOF was age dependent. One acute respiratory failure was not pathology- or TEVAR-related. Three causes of mortality were unknown.

#### Overall survival

The overall survival rates at 12 and 60 months were 75.8% (95% CI = [0.76–0.70]) and 56.6% (95% CI = [0.57–0.50]), respectively (Figure 2A). The log-rank comparison indicated a clearly reduced survival rate in groups of emergent compared with elective/urgent patients (p<0.001; Figure 2B). Reduction in survival occurred in-hospital and in the short term. Subgroup comparison recognized reduced overall survival of patients due to ABF and AEF compared with any other indications (log-rank p<0.001 comparing all pathologies; Figure 2C).

#### Incidence of rupture

**Figure 3.**
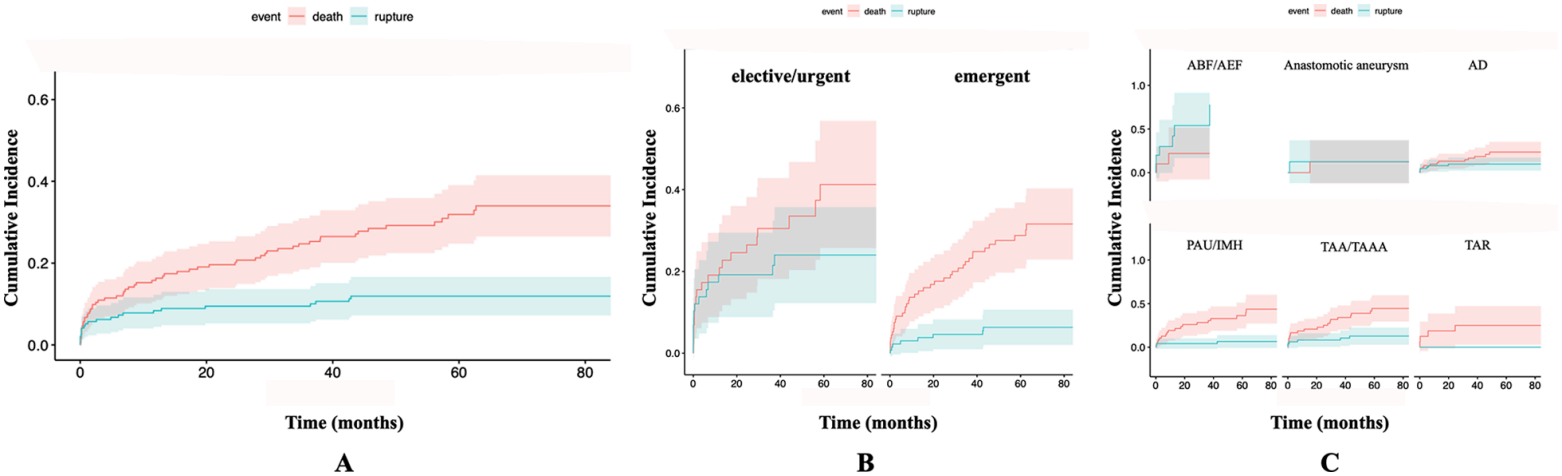
Cumulative incidence of rupture (A) of all patients included in the study, (B) stratified by emergent compared with elective/urgent patients, and (C) stratified by indication. ABF, aortobronchial fistula; AD, aortic dissection; AEF, aortoesophageal fistula; IMH, intramural hematoma; PAU, penetrating aortic ulcer; TAA, thoracic aortic aneurysm; TAAA, thoracoabdominal aortic aneurysm; TAR, traumatic aortic rupture.

Cumulative incidence for rupture at 12, 60, and 90 months was 8.4% (95% CI = [0.04–0.12]), 11.9% (95% CI = [0.07–0.17]), and 11.9% (95% CI = [0.07–0.17]), respectively (Figure 3A). Comparison indicated higher rupture rates in groups of emergent compared with elective/urgent patients (Gray’s test, p<0.001; Figure 3B). A higher rate of ruptures was observed in patients due to ABF and AEF compared with another indication (Gray’s test, p<0.001, comparing all pathologies; Figure 3C).

#### Aortic-related reinterventions

In the midterm, 14 (7.2%, 14/194) ARRs were performed (Table 5). All LSA occlusions (2.1%, 4/194) were due to symptomatic EL type II associated with aortic expansion (Table 5). Three distal extensions were performed to cover distal entry tear with retrograde perfusion of false lumen by progression of chronic expanding aortic dissection (CEAD). Two patients were operated with EVAR: 1 due to expansion of the abdominal part of TAAA and 1 due to isolated abdominal aortic aneurysm (1.0%, 2/194; Table 5). One patient showed symptomatic steal syndrome, and 1 had upper extremity claudication after overstenting of LSA. In both cases, carotid subclavian bypass was performed (Table 5). One transfemoral angiography was performed to exclude EL type II, and 1 visceral bypass stenosis was treated with percutaneous transluminal angioplasty (PTA; Table 5). One open thoracic conversion was performed 25 months after TEVAR due to EL type Ia associated with endograft migration. No conversions were performed after 25 months FU in the midterm, and there were none in the long term.

**Table 5.** Aortic-Related Reinterventions in the Midterm and Long-Term Follow-Up.

Type of reintervention	Overall n = 15	Midterm n = 14	Long term n = 1
Endolining/extension	2.1 (4)	1,5 (3)	
LSA-plug/coiling	2.1 (4)	2.1 (4)	
EVAR	0.5 (1)	1.0 (2)	
Carotid subclavian bypass	1.0 (2)	1.0 (2)	
Diagnostic angiography	0.5 (1)	0.5 (1)	
Conversion	0.5 (1)	0.5 (1)	
PTA of visceral bypass/periscope	1.0 (2)	0.5 (1)	0.5 (1)

Categorical data are presented as absolute numbers and percentage (n = 194).

Abbreviations: EVAR, endovascular aortic repair; LSA, left subclavian artery; PTA, percutananeous transluminal angioplasty.

One patient underwent PTA for stenosis of the periscope endograft 92 months after TEVAR. Primarily the patient received TEVAR due to ruptured TAA with overstenting of the celiac trunk and periscope implantation in the superior mesenteric artery (Table 5).

**Figure 4.**
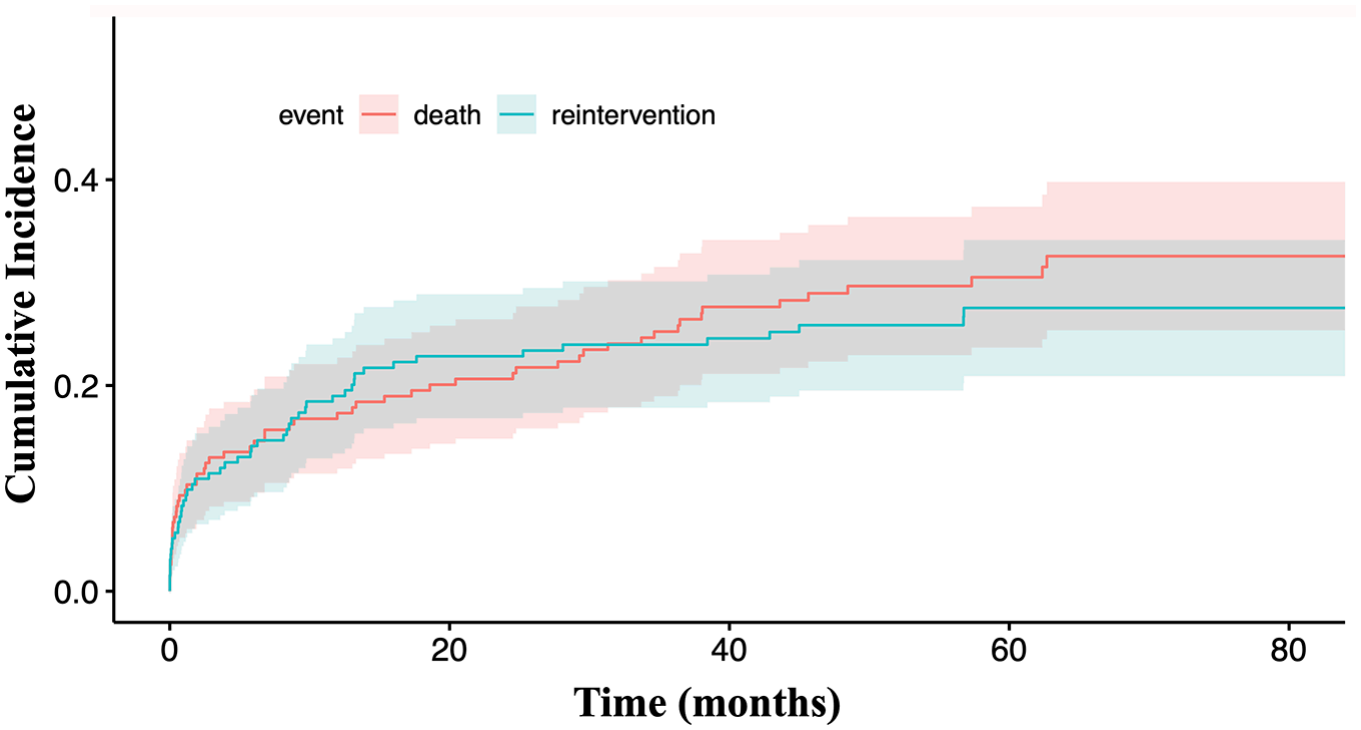
Cumulative incidence of aortic-related reintervention.

Cumulative incidence of ARR was 19.0% (95% CI = [0.13–0.25]), 27.5% (95% CI = [0.21–0.34]), and 27.5% (95% CI = [0.21–0.34]) at 12, 60, and 90 months of FU, respectively (Figure 4). The median time to ARR was 6 months (range: 0–95 months).

#### Procedure-related complications

Overall migration rate was 2.6% (5/194; Table 6). In the midterm FU, 4 migrations (2.1%, 4/194) were registered. In the long term, 1 patient was reported to have migration (0.5%, 1/194). All migrations occurred secondary to aortic expansion due to primary EL type II or disease progression. All migrations were complicated by EL type I/III and/or aortic rupture. Two reinterventions were performed due to migration. One patient rejected the reintervention and died from rupture. One distal extension was failing and resulted in mortality. Two conversions were suggested to be due to EL type Ia, and 1 of them was performed on 25 months after TEVAR (Table 6).

**Table 6.** Case-by-Case Device Migration in the Midterm and Long-Term Follow-Up.

Case	Migration site	Pathology	Postoperative month of migration	Cause of migration	Complication of migration	Reintervention	Outcome
1	Overlapping zone	TAA	12	EL II	EL III	Endolining	Death in 50 months after TEVAR due to larynx carcinoma
2	Proximal landing zone	SATBAD	19	EL II	EL Ia expansion	Conversion on 25 months after TEVAR	Alive 43 months after TEVAR
3	Proximal landing zone	CETBAD	31	EL II	EL Ia expansion	Conversion suggested	Alive 100 months after TEVAR
4	Distal landing zone	TAA	43	EL II	EL Ib with rupture	Extension attempt	Death on the day of reintervention
5	Overlapping zone	TAA	64	Disease progression	Expansion with rupture	Patient rejected reintervention	Death in 91 months after TEVAR

Abbreviations: CETBAD, chronic expandable type B aortic dissection; EL, endoleak; SATBAD, subacute type B aortic dissection; TAA, thoracic aortic aneurysm; TEVAR, thoracic endovascular aortic repair.

**Figure 5.**
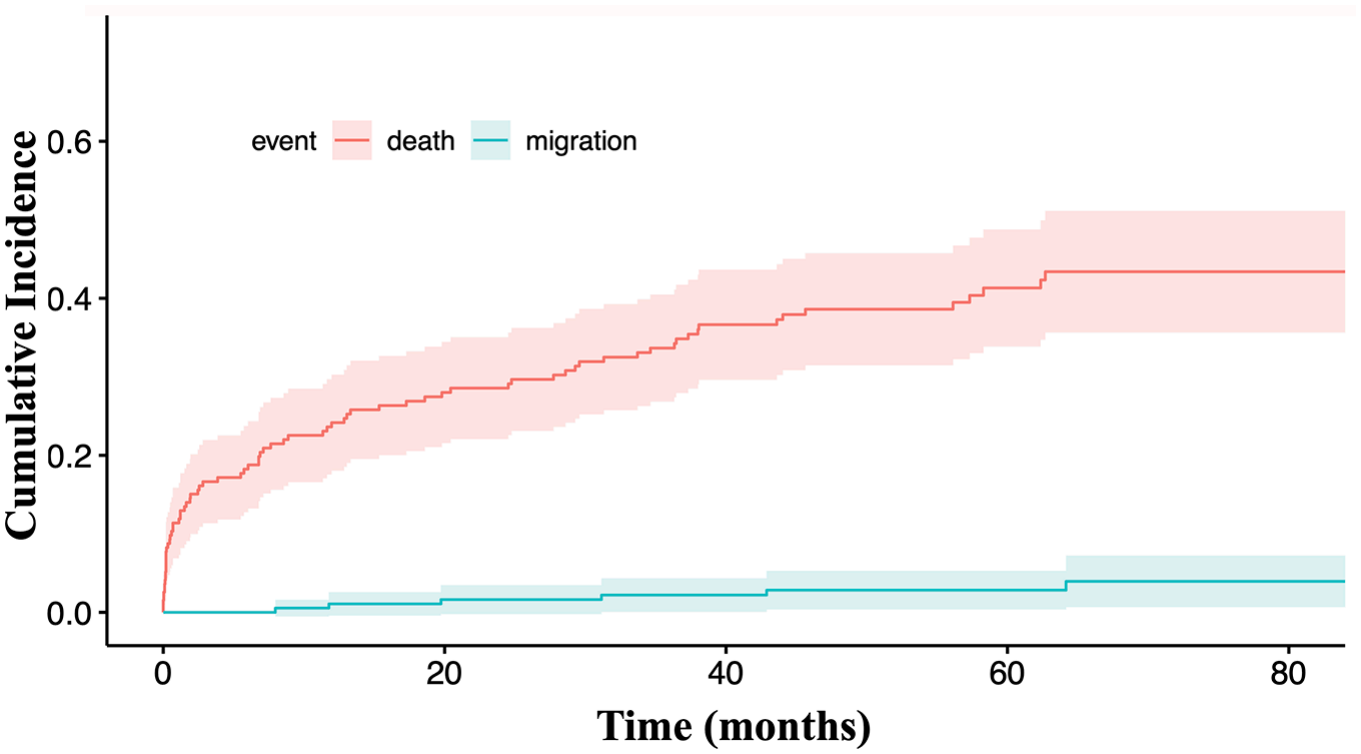
Cumulative migration rate.

Cumulative migration rates were 1.1% (95% CI = [0–0.034]), 2.8% (95% CI = [0.004–0.053]), and 3.9% (95% CI = [0.007–0.072]), at 12, 60, and 90 months, respectively (Figure 5).

Secondary endograft infections, material degradation, or device obstructions were not observed in the midterm or long-term FU.

## Discussion

This study reports real-world single-center experience using the CTAG, with a maximal FU of 8 years (127.8 months) and a median FU of 43.5 months (Q1-Q3: 8.6–67.0), wherein 91.2% (177/194) of patients were followed up to the endpoint and a small number of patients were lost to FU (8.7%, 17/194). Of the patients, 72.2% (140/194) were followed in the midterm, while 29.4% (57/194) were followed over 60 months after TEVAR.

Cambria and colleagues reported a maximum FU period of 3.5 years after TEVAR using CTAG, wherein just 30% (15/50) of patients were followed after 36 months.^7^ The European CTAG registry reported a maximum FU of 2.7 years.^9^ The GREAT registry, which combined the results of treatment using TAG and CTAG, reported a mean FU of 26 months, where only 12.9% (34/264) of patients were followed after 2.7 years and 0.3% (1/264) were followed up to 5 years.^19^ Thus, the current study reports on the largest cohort of patients followed up in the midterm and long term after TEVAR using the CTAG device.

Earlier studies using the CTAG showed good early and midterm rupture-free survival. Jordan and colleagues reported on a rupture-free survival following elective TEVAR due to TAA beyond 90% over 3 years, but only 28% of patients (18/64) were followed up to this timepoint.^6^ The current study showed similar results at 2 years of FU in terms of cumulative incidence (9.5%, 95% CI = [5.3–13.6]) with a larger cohort of patients (128/194). Furthermore, this study showed persistent cumulative incidence for rupture of 12% in late midterm and long-term FU periods.

The considerably higher cumulative incidence of rupture in emergent patients compared with those who underwent elective/urgent TEVARs (Gray’s test, p<0.0001) was due to in-hospital and short-term mortality related to the complexity of the treated pathologies. In the midterm and long term following CTAG, elective group showed improved cumulative incidence for rupture compared with the urgent/emergent group, considering that both groups presented good late midterm and long-term clinical durability of TEVAR using CTAG.

The European CTAG multicenter registry failed to find a reduced survival rates in urgent versus elective treated patients at 2 years of FU; however, this registry had a smaller cohort (n = 100), did not include ABF and AEF cases, and included fewer emergent cases compared with this current study (33% vs 43.8%, respectively).^9^

The current study also observed reduced midterm survival after treatment of AEF compared with other indications (log-rank p<0.0001), with no patients surviving in the long term, which is consistent with data from other trials.^20,21^ However, these TEVAR results in primary septic settings were not device-related. For TAA, PAU, IMH, and TAR, CTAG showed a similar cumulative incidence for rupture in midterm and long-term FU.

The majority of ARRs after TEVAR were performed in the early- and midterm.^22^ Most reinterventions were related to EL or the pathology.^7,20^ Previously published studies reported a 2-year reintervention-free survival rate of 53% associated with CTAG, similar to the midterm results observed with other types of contemporary endografts.^9,21,23^ The current study was generally in line with these outcomes; however, we present a superior 2-year ARR cumulative incidence of 22.8% (95% CI = [0.17–0.29]). Moreover, the current study demonstrates the persistent long-term durability of CTAG in regard to the rate of reinterventions. After 60 months of FU, cumulative incidence for reintervention was over 27.5% (95% CI = [21–0.34]), with a similar durability up to 90 months of FU.

Reinterventions are associated with device migration and ELs.^20,24^ Previously published series reported migration rates up to 8% with contemporary aortic endografts.^24,25^ Geisbüsch reported a 7.3% (9/123) endograft migration rate requiring reintervention in 44% of these migration cases.^24^ A study by Jordan and colleagues showed a 2% (1/50) migration rate without the need for reintervention.^6^

This recent CTAG study showed a very low migration rate in the midterm and long term (2.6%, 5/194) after the 8 years following TEVAR, considering that all late migrations occurred secondary to aortic expansion due to persistent EL type II and were not device-related.

A single conversion due to CTAG infection following pneumonia in the midterm was reported by Böckler et al.^9^ A CTAG dissection trial showed no conversions due to DRCs in the midterm.^7^ The results of this study were in line with these findings, showing very low conversion rates in the midterm and long-term FU (1.0%, 2/194).

Moreover, no new endograft infections, material degradation, or device obstructions were presented. Thus, the findings of the current study suggest very low DRC rates for CTAG in the midterm and long-term FU.

## Limitations

This study is limited due to the retrospective design without control group with other commercially available stent grafts. Heterogeneous indications and high rate of emergencies for treatment may introduce bias regarding the length of FU and reintervention rates. In addition, the small size for subgroup analysis might lead to measurement bias. Few patients were followed more than 60 months after TEVAR, which may introduce bias in the reporting of long-term outcomes.

## Conclusions

The herein reported 10-year real-world single-center experience with the CTAG observed favorable long-term outcome. Thus, the device demonstrates appropriate persistent safety, efficacy, and clinical durability up to long-term FU in the treatment of diverse thoracic aortic pathologies.
